# Unmet need for chronic mental ill health: A population-based record linkage study

**DOI:** 10.23889/ijpds.v4i1.1122

**Published:** 2019-11-29

**Authors:** M Rosato, F Tseliou, D O’Reilly

**Affiliations:** 1 Bamford Centre for Mental health and Wellbeing, Ulster University, BT48 7JL, Londonderry, Northern Ireland; 2 Centre for Public Health, Queen’s University Belfast, BT12 6BA, Belfast, Northern Ireland

## Abstract

**Introduction:**

Many people with chronic mental ill health do not receive the treatment they require, though the true extent of the associated socio-demographic and socio-economic factors is unknown.

**Objectives:**

This unique record linkage study quantifies the characteristics of those reporting chronic poor mental health and the likelihood of being in receipt of pharmacological treatment for those who report chronic mental ill health.

**Methods:**

The Northern Ireland Longitudinal Study (NILS), a random 28% of the 2011 Census returns (aged 25-74), was linked to a population-wide electronic database of prescribed medications. All cohort attributes, including presence of chronic poor mental health were derived from the Census. Logistic regression was used to test the likelihood of people with poor mental health being prescribed psychotropic medication. These findings were compared against similarly derived characteristics of those with respiratory illness on treatment.

**Results:**

Overall, 23,803 (8.3%) of the enumerated 286,717 reported poor mental health and, while 81.5% received pharmacological treatment, those of non-white background (OR=0.38: 95%CI=0.26-0.54), never married (OR=0.67: 95%CI=061-0.73), unemployed (OR=0.65: 95%CI=0.53-0.81) or living in a rural area (OR=0.88: 95%CI=0.79-0.98) were less likely than their respective peers to receive medication for poor mental health. Non-treatment of respiratory illness was less socially patterned.

**Conclusions:**

Some but not all of the observed variation in receipt of psychotropic medicines may represent unmet need. Further studies are required to clarify the patterning of and possible reasons for underuse, including understanding of and attitudes towards healthcare services of groups who are identified as being less likely to receive treatment (for example ethnic minorities and unemployed).

**Highlights:**

- Self-reported chronic mental ill health varied by socio-demographic and socio-economic characteristics.
- Although a high proportion of people received psychotropic medication for their mental ill health, there were evident social patterns among those who do not receive treatment.
- Ethnic minorities, those whose were not married or unemployed were considerably more likely to experience unmet need.

## Introduction

It is generally accepted that a significant proportion of people with mental ill health are under-diagnosed and untreated in primary care settings [1,2]. The presence of unmet need referring to a treatment gap between poor mental health status and treatment received by individuals with mental health problems has been previously highlighted at a United Kingdom [3,4] and Europe wide level [5]. There is a range of societal, cultural and individual factors that can influence treatment practices and lead to under-treating individuals. This could be linked to help-seeking behaviours, with men [6,7] and people residing in rural settings [8] being less likely to seek treatment. A similar effect has also been observed for ethnic minorities [9] and people living in socially deprived areas [4]. When focusing on medication rates, lower uptake of psychotropic prescriptions such as antidepressants has been identified among individuals of low educational and socio-economic status [10]. It is important to note that low patterns of psychotropic medication can lead to higher rates of distress due to untreated mental ill health problems [11].

However, the current rates of medication prescription for mental ill health across the world have recently become the subject of some debate. Perceptions have shifted suggesting that people with poor mental health are more likely to be over than under-medicated, though this varies across different healthcare systems. Reports of over-treatment [12] from some parts of the world such as the United States have called attention to the higher than recommended levels of medications and especially antidepressants [13]. Over-treatment can disproportionately affect individuals with mild to less severe symptoms, as they are more likely to receive antidepressants due to insufficient time for provision of alternative treatment options [14], despite the fact that medication might not be either recommended [15] or considered efficient treatment for those patients [16]. At population level, practices of over-prescription can potentially lead to worsening mental health problems instead of alleviating them [12].

In that context, the ability of general practitioners (GPs) to identify individuals with mental health conditions presenting in primary care settings, can potentially lead to either over- or underestimations of mental ill health, as 90% of individuals with mental health problems are managed within primary care settings [17]. Consequently, the accuracy of clinical diagnoses of mental health conditions in primary care can affect the likelihood of receiving treatment as GPs’ accuracy in identifying cases has been shown to vary [18].

Despite the potential impact of variations in medication practices, with both under-treatment and over-prescription being highlighted in the literature, there is still a lot of uncertainty stemming from methodological limitations of previous studies. With the exception of a number of Scandinavian studies [19,20], many earlier studies have been unable to accurately measure the level of treatment received on a community level. Additional issues include the use of self-reported measures of medication uptake [21] that might cause issues due to recall bias, non-representative samples linked to non-response rates [22] and cross-sectional study designs [5] that can only provide a snapshot of the population at one time-point. It has been further proposed that a more appropriate assessment of mental ill health would account for both the presence of a mental health condition and its overall impact on the individual [23].

If we make the assumption that prescription of psychotropic medications is the predominant form of treatment for mental illness, at least in Northern Ireland, then dissonances between those who report chronic poor mental health and those who receive psychotropic medications may indicate subgroups in the population whose needs are not being addressed by current approaches. The use of linked administrative data related to need and treatment across the entire population can overcome methodological hurdles and provide an indicator of areas of possible unmet need.

In this paper we therefore have two aims; (i) to establish the proportions and demographic and socio-economic characteristics of individuals reporting chronic poor mental health and (ii) for those with chronic poor mental health, to quantify the factors related to not being in receipt of psychotropic medication.

## Methods

### Data Sources

This study utilised data drawn from the Northern Ireland Longitudinal Study (NILS), which was formed in 2001 with a representative 28% sample of the NI population and is maintained by the Northern Ireland Statistics and Research Agency (NISRA). It comprises a linkage of the Northern Ireland Health Card Registration database (ensuring universal and free at-the-point-of-service healthcare) and Census returns, to which routinely collected vital statistics events such as births, deaths and migrations are linked on a regular basis. A description of the full cohort and the linkage methodology that was implemented is available elsewhere [24]. The NILS cohort was linked to a population-wide prescribing dataset through the use of the Health and Care Number, a unique identifier enabling linkage to other health service databases, including the centralised collation of primary care prescriptions (see below).

### Study population

The study compared self-reports of chronic mental ill health from the 2011 Census returns (March 27th of that year) to psychotropic prescribing uptake for enumerated individuals in the twelve months following the Census. A total of 37,342 NILS members aged 16 and over were enumerated in the 2011 Census. People living in institutional settings (N=4,836) and those with missing data on the health conditions question (N=30,638) were excluded. For this analysis, a subset of 286,717 NILS members aged between twenty-five and seventy-four years at Census were selected as these represent the population group for whom socio-economic status can be reasonably stated (excluding those aged >25 & <74 years: N=56,151). All personal characteristics were drawn from the Census: these include age (in ten-year age-bands), sex and marital status (grouped as married; never married; and a single group classifying those widowed, separated and divorced). Northern Ireland is ethnically relatively homogenous, so only two ethnic groups were feasible (white; non-white). Additional socio-economic and area-level factors thought to be associated with mental health stigma were also included. Socio-economic status was determined using several indicators from the Census: these include educational attainment (grouped as no formal qualifications, intermediate level, degree-level); occupational social class derived using the National Statistics Socio-economic Classification (NS-SEC) [25] – with categories as shown in Table 1; housing tenure (owner-occupation, renting) and household car availability (two or more cars, one, no cars).

### Assessment of chronic ill health in the Census

The 2011 Census included a question asking about the presence of *“any of the following conditions which have lasted, or are expected to last, at least 12 months?”* Respondents were asked to tick all applicable conditions from a list of ten (ranging from shortness of breath to sensory problems). For this study, we examined those respondents who ticked the category *“an emotional, psychological or mental health condition (such as depression or schizophrenia)”*. For brevity, we term this a chronic mental health problem. This measure has been used in a number of studies [26] providing similar estimates of population mental health with standardised measures such as the General Health Questionnaire (GHQ) and demonstrating a high degree of validity [27]. We also examined responses to another group - *“shortness of breath or difficulty breathing (such as asthma)”* - as a specificity comparison group for those seeking or receiving treatment for a respiratory problem (See Appendix). Breathing difficulty was chosen as it is quite common in the general population, can present across different age groups and is not considered a stigmatised condition. Although the 2011 Census included other chronic health conditions, these would not allow for a comparison between self-reported health conditions and medication uptake, as most physical conditions included are not treated pharmacologically at the point of contact (for example visual or hearing difficulties).

### Area characteristics

Area-level characteristics were assessed using: (i) a measure of population density based on the NISRA Classification of Settlements [28], grouping individuals as living in urban, intermediate and rural settlements of >75,000 people, 2,500-75,000 and < 2,500 people respectively and (ii) area-level deprivation based on uptake of means-tested social security benefits [29] and calculated for 890 super output areas (SOA), each with an average population of approximately 2,000 people and subsequently grouped into quintiles ranging from least to most deprived.

### Medication prescription

In Northern Ireland, the National Health Service provides universal health coverage free at the point of use, including prescriptions. Medications prescribed by GPs and dispensed from community pharmacies are (since 2009) collated centrally in an Enhanced Prescribing Database (EPD). This comprises of data on prescribed medicines, including information on the name of the dispensed medicine, its British National Formulary (BNF) category (a standard United Kingdom referencing system) and the date it was dispensed. For this study information on four categories of psychotropic medication was extracted: anxiolytic and antidepressant medications (BNF categories 4.1.2 and 4.1.3 respectively), anti-psychotic medication (oral and depot; BNF categories 4.2.1 and 4.2.2) and anti-mania medications (BNF categories 4.2.3). We also extracted information on respiratory medication (BNF Chapter 3, excluding antihistamine medication) to examine the uptake of medication for a common non-stigmatising physical health condition (in this case breathing difficulties) (See Appendix). This would act as a comparator to the receipt of psychiatric medication and help differentiate the socio-demographic correlates of stigma, which would be found only with the mental health medications from other explanations of low uptake, such as lower attendance at GPs or lack of knowledge of the system, which should be common to both types of medication. Mediation prescription use was assessed by summing the monthly usage, to include receipt of any prescription for medication thus covering every month during the twelve-month period following the Census. For depot injections, at least one such prescription during the same period was deemed sufficient.

NILS and the EPD were linked through the NI Health and Care Number by the data custodians, with all key matching fields and other potential identifiers removed before being made available to the researchers. All analysis was carried out within the NISRA secure setting. The study was approved by the Office for Research Ethics Committees Northern Ireland.

### Analysis strategy

Descriptive statistics were used to describe the characteristics of the study population, as well as, the link between these characteristics and reporting a chronic mental health problem in the Census. These included individual (age, sex, ethnicity, marital status, education, social class), household (housing tenure, household car access) and area (locale of residence, area-level deprivation) level factors. Among those individuals that reported chronic mental ill health, variations in the receipt of a psychotropic medication prescription in the following twelve months was also explored in relation to these characteristics. Logistic regression models were implemented to investigate how the likelihood of reporting chronic mental ill health varied by the aforementioned population characteristics. Two steps of logistic regression models were presented, namely: i) a model specifying all population characteristic separately, each minimally adjusting for age/sex and ii) a fully adjusted model, including all population characteristics described above. These two model structures were repeated to explore the characteristics associated with the likelihood of receiving a prescription in the twelve-month period among those reporting a chronic mental health condition. As both seeking and receiving pharmacological treatment have been shown to vary between individuals presenting with physical versus mental ill health, the analysis was repeated using chronic respiratory conditions as the outcome of interest (See Appendix). This provided a comparison aiming to identify whether uptake variation by socio-demographic characteristics was observed across individuals with physical and mental ill health.”

## Results

Our cohort consisted of 23,803 individuals (8.3% of study population) who reported that they had a long-standing mental, emotional or psychological problem at the time of the Census. Table 1 shows that prevalence rates of chronic mental ill health were higher among individuals who were middle-aged (45-54 years: 10.6% and 55-64 years: 10.1%), female (9.5% versus male: 7.0%), of white ethnicity (8.4% versus non-white: 3.3%), separated/divorced/widowed (16.5% versus married: 5.5%) and with no academic qualifications (13.8%). In terms of household characteristics, lower social class (except for those unemployed: 8.3% versus never worked: 24.8%) as well as other socio-economic factors such as being in rented accommodation (16.7% versus owner-occupiers: 5.6%) and having no access to a car (20.3% versus ≥2 cars: 4.1%) were linked to increased rates of reporting chronic poor mental health in the Census. A gradient association was observed between reporting chronic mental ill health and the level of area deprivation with the highest proportion being identified among those residing in the most deprived areas (14.2% versus least deprived:4.6%).

After adjustment for a range of individual, household and area-level factors, older individuals were less likely than younger individuals to report the presence of a chronic mental health condition at the time of the Census (65-74 years: OR=0.80: 95%CI=0.75-0.85), though that was not the case during the initial model (OR=1.00: 95%CI=0.95-1.06) suggesting the presence of a complex interplay between a range of socio-demographic factors and disclosure of chronic mental ill health. Mixed results were also observed in relation to the association between social class and chronic mental health status, with those unemployed shifting from being twice as likely as those in managerial positions to report mental ill health (OR=2.00: 95%CI=1.82-2.20) to being less likely to do so after full adjustment (OR=0.69: 95%CI=0.62-0.76). Those of non-white ethnic background were significantly less likely to report poor mental health (OR=0.38: 95%CI=00.32-0.45). People in rural and intermediate areas were less likely than their urban dwelling peers to report poor mental health but on further adjustment including adjustment for housing tenure and car ownership, the urban/rural differences were greatly attenuated.

**Table 1: Characteristics of persons 25-74 and likelihood of reporting mental health problems at Census. Data represent numbers (percentages) and Odds Ratios (95% Confidence Intervals) from logistic regression models.** ^$^ fully adjusted model, adjusted for all variables listed in column one – age, sex, ethnicity, marital status, highest educational qualification, tenure, car access, locale and area-level deprivation measures

		No prescription in the 12 months following Census	Received prescription in the 12 months following Census	Rates of individuals receiving prescription in the 12 months following Census	Adjusted: age & sex	Fully Adjusted^$^
		N(%)	N (%)	%	OR (95% CI)	OR (95% CI)

Cohort		4,412 (100.0)	19,391 (100.0)	81.50%		

Age	25-34	946 (21.4)	2,567 (13.2)	73.10%	1.00	1.00
	35-44	1,047 (23.7)	4,643 (23.9)	81.60%	1.65 (1.49-1.82)	1.55 (1.39-1.72)
	45-54	1,121 (25.4)	5,930 (30.6	84.10%	2.02 (1.83-2.23)	1.82 (1.63-2.03)
	55-64	872 (19,8)	4,450 (22.9)	83.60%	1.99 (1.79-2.21)	1.71 (1.51-1.93)
	65-74	426 (9.7)	1,801 (9.4)	80.90%	1.64 (1.44-1.86)	1.37 (1.18-1.58)

Sex	Male	2,284 (51.8)	7,336 (37.8)	76.30%	1.00	1.00
	Female	2,128 (48.2)	12,055 (62.2)	85.00%	1.81 (1.70-1.94)	1.78 (1.66-1.90)

Ethnicity	White	4,360 (98.8)	19,314 (99.6)	81.60%	1.00	1.00
	Non-white	55 (0.2)	77 (0.4)	59.70%	0.36 (0.26-0.52)	0.38 (0.26-0.54)

Marital status	Married	1,502 (34.0)	7,837 (40.4)	83.90%	1.00	1.00
	Never married	1,643 (37.2)	5,313 (27.4)	76.40%	0.76 (0.69-0.82)	0.67 (0.61-0.73)
	Sep-Div-Wid	1,267 (28.8)	6,241 (32.2)	83.10%	0.86 (0.79-0.94)	0.75 (0.68-0.82)

Education	Degree-level	821 (18.6)	3,097 (16.0	79.10%	1.00	1.00
	Intermediate	1,718 (38.9)	7,195 (37.1)	80.70%	1.13 (1.03-1.25)	1.06 (0.96-1.17)
	No qualifications	1,873 (42.5)	9,099 (46.9)	82.90%	1.25 (1.13-1.37)	1.14 (1.02-1.27)

Social class/Economic activity	Managerial/Prof	776 (17.6)	3,065 (15.8	79.80%	1.00	1.00
	Intermediate	608 (13.8)	2,735 (14.1)	81.80%	1.11 (0.98-1.25)	1.07 (0.95-1.22)
	Own account	267 (6.0)	912 (4.7)	77.40%	1.02 (0.87-1.20)	0.96 (0.81-1.13)
	Semi-routine	1,119 (25.4)	5,440 (28.1)	82.50%	1.22 (1.10-1.35)	1.13 (1.01-1.26)
	Routine	839 (19.0)	4,243 (21.9)	83.50%	1.30 (1.17-1.45)	1.16 (1.03-1.32)
	Never worked	599 (13.5)	2,466 (12.7)	80.50%	1.10 (0.97-1.24)	1.01 (0.88-1.16)
	Unemployed	153 (3.5)	369 (1.9)	70.70%	0.69 (0.56-0.85)	0.65 (0.53-0.81)
	Student	51 (1.2)	161 (0.8)	75.90%	0.87 (0.63-1.21)	0.84 (0.60-1.17)

Housing tenure	Owner	205,096 (78.0)	12,242 (51.4)	5.60%	1.00	1.00
	Renting	57,818 (22.0)	11,561 (48.6)	16.70%	3.72 (3.62-3.83)	1.75 (1.69-1.81)

Household car access	Two or more cars	136,957 (52.1)	5,906 (24.8)	4.10%	1.00	1.00
	One car	95,006 (36.1)	10,022 (42.1)	9.50%	2.62 (2.54-2.71)	1.69 (1.62-1.75)
	No car	30,891 (11.8)	7,875 (33.1)	20.30%	6.42 (6.19-6.66)	2.37 (2.26-2.48)

Locale of residence	Urban	90,917 (34.6)	9,677 (40.7)	9.60%	1.00	1.00
	Intermediate	81,731 (31.1)	7,732 (32.5)	8.70%	0.89 (0.86-0.92)	0.95 (0.92-0.98)
	Rural	58,298 (22.2)	3,798 (16.0)	6.10%	0.61 (0.59-0.63)	0.98 (0.93-1.02)
	Missing	31,968 (12.1)	2,596 (10.8)	7.50%	0.79 (0.75-0.83)	1.05 (1.00-1.10)

Area-level deprivation (SOA)	Least deprived	52,903 (20.1)	2,580 (10.8)	4.60%	1.00	1.00
	Less deprived	56,497 (21.5)	3,703 (15.6)	6.20%	1.37 (1.30-1.45)	1.12 (1.06-1.18)
	Average	53,841 (20.5)	4,248 (17.8)	7.30%	1.67 (1.59-1.76)	1.18 (1.12-1.24)
	More deprived	52,116 (19.8)	5,647 (23.7)	9.80%	2.31 (2.20-2.43)	1.28 (1.22-1.35)
	Most deprived	45,501 (17.3)	7,497 (31.5)	14.20%	3.56 (3.40-3.73)	1.36 (1.29-1.43)
	Missing	1,796 (0.8)	128 (0.6)	6.70%	1.53 (1.27-1.83)	1.06 (0.87-1.29)

A total of 19,391 (81.5%) cohort members were in receipt of psychotropic medication in the twelve months following the Census, leaving 18.5% with no prescription for their chronic mental health problems. Of those reporting chronic mental ill health, the higher uptake of pharmacological treatment in the following period was observed among females rather than males (85.0% and 76.3% respectively) and the middle-aged (45-54: 84.1% and 55-64: 83.6% respectively) compared to younger people (25-34: 73.1%). Although those in the 65-74 year age-group were less likely to report poor mental health, they were still more likely to receive medication in comparison to younger individuals (OR=1.37: 95%CI=1.18-1.58), potentially suggesting variations in presentation and reporting by patients or prescribing propensity by GPs. Lower psychotropic medication uptake was observed among the never married (76.4% versus married: 83.9%) who, along with the separated/widowed/divorced, were less likely to be in receipt of medication than those married (OR=0.67: 95%CI=0.61-0.73; and OR=0.75: 95%CI=0.68-0.82 respectively). This is in contrast to the higher rates of self-reported mental ill health among those two groups, suggesting an imbalance between need and treatment. Furthermore, much lower rates of pharmacological treatment were identified among individuals with a non-white background when compared with those of white ethnic background (59.7% and 81.6% respectively) which, after further adjustment for potential confounders, equated to an odds ratio of 0.38 (95%CI=0.26-0.54).

The relationship between prescribing and socio-economic status was not straightforward, though generally higher for those in the lower social strata. Those who were unemployed were also less likely to be prescribed psychotropic medication in comparison to those of managerial status (OR=0.65: 95%CI=0.53-0.81), while those in semi-routine and routine social classes (OR=1.13: 95%CI=1.01-1.26 and OR=1.16: 95%CI=1.03-1.32 respectively) were more likely than those in professional classes to be receiving medication. Prescription rates were also higher for those in rented accommodation than those in owner occupation (OR=1.28: 95%CI=1.18-1.38). Finally, the analysis showed that patients from rural areas were less likely to be in receipt of prescriptions when compared to urban areas (OR=0.88: 95%CI=0.79-0.98), with no difference between intermediate areas and urban residents.

**Table 2: Factors associated with the likelihood that people reporting mental health problems at Census receiving psychotropic prescription in the following 12 months. Data represent numbers (percentages) and Odds Ratios (95% Confidence Intervals) from logistic regression models.** ^$^ fully adjusted model, adjusted for all variables listed in column one – age, sex, ethnicity, marital status, highest educational qualification, tenure, car access, locale and area-level deprivation measures

		No prescription in the 12 months following Census	Received prescription in the 12 months following Census	Rates of individuals receiving prescription in the 12 months following Census	Adjusted: age & sex	Fully Adjusted^$^
		N(%)	N (%)	%	OR (95% CI)	OR (95% CI)

Cohort		4,412 (100.0)	19,391 (100.0)	81.50%		

Age	25-34	946 (21.4)	2,567 (13.2)	73.10%	1.00	1.00
	35-44	1,047 (23.7)	4,643 (23.9)	81.60%	1.65 (1.49-1.82)	1.55 (1.39-1.72)
	45-54	1,121 (25.4)	5,930 (30.6	84.10%	2.02 (1.83-2.23)	1.82 (1.63-2.03)
	55-64	872 (19,8)	4,450 (22.9)	83.60%	1.99 (1.79-2.21)	1.71 (1.51-1.93)
	65-74	426 (9.7)	1,801 (9.4)	80.90%	1.64 (1.44-1.86)	1.37 (1.18-1.58)

Sex	Male	2,284 (51.8)	7,336 (37.8)	76.30%	1.00	1.00
	Female	2,128 (48.2)	12,055 (62.2)	85.00%	1.81 (1.70-1.94)	1.78 (1.66-1.90)

Ethnicity	White	4,360 (98.8)	19,314 (99.6)	81.60%	1.00	1.00
	Non-white	55 (0.2)	77 (0.4)	59.70%	0.36 (0.26-0.52)	0.38 (0.26-0.54)

Marital status	Married	1,502 (34.0)	7,837 (40.4)	83.90%	1.00	1.00
	Never married	1,643 (37.2)	5,313 (27.4)	76.40%	0.76 (0.69-0.82)	0.67 (0.61-0.73)
	Sep-Div-Wid	1,267 (28.8)	6,241 (32.2)	83.10%	0.86 (0.79-0.94)	0.75 (0.68-0.82)

Education	Degree-level	821 (18.6)	3,097 (16.0	79.10%	1.00	1.00
	Intermediate	1,718 (38.9)	7,195 (37.1)	80.70%	1.13 (1.03-1.25)	1.06 (0.96-1.17)
	No qualifications	1,873 (42.5)	9,099 (46.9)	82.90%	1.25 (1.13-1.37)	1.14 (1.02-1.27)

Social class/Economic activity	Managerial/Prof	776 (17.6)	3,065 (15.8	79.80%	1.00	1.00
	Intermediate	608 (13.8)	2,735 (14.1)	81.80%	1.11 (0.98-1.25)	1.07 (0.95-1.22)
	Own account	267 (6.0)	912 (4.7)	77.40%	1.02 (0.87-1.20)	0.96 (0.81-1.13)
	Semi-routine	1,119 (25.4)	5,440 (28.1)	82.50%	1.22 (1.10-1.35)	1.13 (1.01-1.26)
	Routine	839 (19.0)	4,243 (21.9)	83.50%	1.30 (1.17-1.45)	1.16 (1.03-1.32)
	Never worked	599 (13.5)	2,466 (12.7)	80.50%	1.10 (0.97-1.24)	1.01 (0.88-1.16)
	Unemployed	153 (3.5)	369 (1.9)	70.70%	0.69 (0.56-0.85)	0.65 (0.53-0.81)
	Student	51 (1.2)	161 (0.8)	75.90%	0.87 (0.63-1.21)	0.84 (0.60-1.17)

Housing tenure	Owner	2,378 (53.9)	9,864 (50.9)	80.60%	1.00	1.00
	Renting	2,034 (46.1)	9,527 (49.1)	82.40%	1.21 (1.13-1.30)	1.28 (1.18-1.38)

Household car access	Two or more cars	1,128 (25.6)	4,778 (24.6)	80.90%	1.00	1.00
	One car	1,836 (41.6)	8,186 (42.2)	81.70%	1.07 (0.99-1.17)	1.06 (0.97-1.16)
	No car	1,448 (32.8)	6,427 (33.1)	81.60%	1.10 (1.00-1.20)	1.09 (0.97-1.23)

Locale of residence	Urban	1,775 (40.2)	7,892 (40.7)	81.60%	1.00	1.00
	Intermediate	1,327 (30.1)	6,360 (32.8	82.30%	1.06 (0.98-1.14)	1.01 (0.93-1.10)
	Rural	755 (17.1)	3,043 (15.7)	80.10%	0.91 (0.82-0.99)	0.88 (0.79-0.98)
	Missing	500 (11.3)	2,096 (10.8)	80.70%	0.99 (0.89-1.11)	0.97 (0.86-1.09)

Area-level deprivation (SOA)	Least deprived	524 (11.9)	2,056 (10.6)	79.70%	1.00	1.00
	Less deprived	682 (15.5)	3,021 (15.6)	81.60%	1.13 (0.99-1.28)	1.09 (0.96-1.25)
	Average	779 (17.7)	3,469 (17.9)	81.70%	1.15 (1.02-1.31)	1.10 (0.97-1.25)
	More deprived	988 (22.4)	4,659 (24.0)	82.50%	1.21 (1.08-1.37)	1.10 (0.97-1.25)
	Most deprived	1,415 (32.0)	6,082 (31.4)	81.10%	1.11 (1.00-1.25)	0.98 (0.87-1.11)
	Missing	24 (0.5)	104 (0.5)	81.50%	1.11 (0.70-1.76)	1.07 (0.67-1.71)

The likelihood of receiving respiratory medication for the cohort of 25,983 people in the Census who reported chronic breathing difficulties was also explored (See Appendix). Overall, 82% of these people received at least one prescription in the following 12 months; this was shown to vary by socio-demographic factors though in a different manner to psychotropic medications. Uptake of respiratory medications exhibited a slightly stronger age gradient, perhaps reflecting the milder and more intermittent breathing difficulties (such as asthma) at younger ages. Prescriptions were also higher amongst females than males, though the difference (OR=1.21: 95%CI=1.15-1.28) was much lower than that observed among individuals with chronic mental ill health. The differences between white and non-white ethnic groups were also much lower for respiratory than for psychotropic medications (OR=0.78: 95%CI-0.56-1.07 and OR=0.38: 95%CI=0.26-0.54 respectively). Furthermore, no socio-economic, household or area-level variations were detected, indicating differing medication uptake patterns between individuals with chronic physical and mental health.

## Discussion

This large population wide study sought to quantify variations in the prevalence of poor mental health as assessed by self-report in the 2011 Census and, through record linkage, to examine variations in treatment levels for those who reported poor mental health, as assessed by uptake of psychotropic medications in the 12 months following the Census. This study confirmed the established socio-demographic correlates and socio-economic gradients in mental health; namely that the likelihood of poor mental health was higher in women, the middle aged, those who were not married and those who were more socially disadvantaged. Self-reported levels of poor mental health were particularly low amongst non-white ethnic minorities (OR=0.38: 95%CI=0.32-0.45).

The present study also demonstrated that most people (82%) reporting chronic mental ill health received at least one prescription for psychotropic medication over the following twelve months. This is perhaps reassuring in that a very high proportion is being treated, but perhaps worrying given the heavy reliance on pharmacological therapy. However, the remaining 18% of people with mental ill health who did not receive any pharmacological treatment did not appear to be randomly distributed; this could be further supported by the fact that the observed social patterns were not present in those not receiving treatment for breathing difficulties. Individuals who were not married, unemployed or were living in rural communities were less likely to receive treatment for poor mental health. Much of this variation might be understood within the current paradigms of social support [30] or variations in primary care consultation rates [31], but as equivalent social patterns of non-treatment of respiratory medications for people reporting breathing difficulties were either non-existent or greatly attenuated (See Appendix), it could be suggested that stigma might also play a significant part [32].

Rates of psychotropic prescriptions were higher in middle-aged individuals when compared to their younger counterparts, which is in accordance with previous reports [33]. However, it is unclear if this indicates under-treatment of young people, perhaps related to lower levels of presentation to primary care services by younger individuals [34] or a higher prevalence of non-pharmacological treatment at younger ages. Treatment rates for mental ill health were also substantially higher for women, perhaps because they are more likely to recognise and report their mental health conditions, and subsequently seek and receive mental health treatment [35]. However, small gender differences were also evident for receipt of respiratory medications (See Appendix), suggesting that differential access to healthcare services may also be important. The lower treatment rates in men is likely to be multifactorial including being less likely to recognise and acknowledge a significant mental health problem and to subsequently seek help [1], experiencing psychosocial barriers linked to stigmatising beliefs about mental ill health, and compounded by lower primary care attendance rates [36].

It is notable that while married individuals were less likely to report chronic mental ill health, married people were more likely than their non-married peers to receive psychotropic medication for their poor mental health. This is surprising given the higher rates of health service utilisation by widowed and divorced people [37]. However, marriage may be associated with a range of health promoting activities, perhaps arising from increased partner pressures or because of the perceived obligation to maintain health or of exposure to wider social influences [30]. This may explain the higher reported levels of cholesterol screening amongst married men and women in the US [38] and of breast screening in women in the UK [39].

The study confirmed the complex association between mental health and social status as medication use was generally and moderately higher among those more socially disadvantaged [10,40], though this was not the case for those who were unemployed at the time of the Census. It may be that the high prevalence of mental ill health reduces stigma and increases likelihood of recognition and propensity to seek treatment, though Holman, using the data from the British Social Attitudes survey, found that the relationship between social disadvantage and stigma was not straightforward and varied by sex and according to the measure of disadvantage [41]. The higher primary care attendance rates of the more disadvantaged [42], which is mostly explained by morbidity rates [43], may also present additional opportunities for diagnosis and treatment, though the alternative explanation that more affluent sufferers may be preferentially seeking and accessing psychological treatments cannot be discounted.

Ethnic minorities, referring to individuals of non-white ethnic background in this study, exhibited different self-reporting and treatment patterns. Although they were only about one third as likely as the white population to report chronic poor mental health (OR=0.38: 95%CI=0.32-0.45), those who were unwell were only about one third as likely to receive psychotropic medications in the follow-up period (OR=0.38: 95%CI=0.26-0.54). However, the interpretation of this is not straightforward and the explanations may be as various as the different constituent groups. On one hand the lower likelihood of treatment could be seen as evidence of the lower rates of healthcare access as previously reported among individuals from Pakistani, Indian and Bangladeshi backgrounds [44] and this is possibly supported by the slightly (though non-significant) lower likelihood of receiving medication for breathing difficulties. It is known that reduced language proficiency can act as a significant barrier to accessing available primary care services, and to receiving an appropriate diagnosis and treatment. This is perhaps more important for mental health issues [45], but unfortunately, proficiency in English was not available for this study. The lower medication rates could also signal the impediment of culturally-related mental health stigma among non-Western ethnic groups such as Chinese patients [32], and this might explain the much lower likelihood of being on psychotropic medications. However, the apparent treatment shortfall could be also be attributable to a greater use of alternative or complimentary medicine rather than traditional pharmacological treatment, which is known to be common amongst individuals from Non-European countries [46,47].

Finally, individuals in rural areas were less likely to be in receipt of pharmacological treatment, potentially highlighting the presence of different medication practices or help-seeking behaviours between residents of rural and urban areas. There is evidence that the prevalence of poor mental health is higher in cities though whether this is due to social drift or factors inherent to cities is unclear [8]. In the current study the prevalence of chronic poor mental health was considerably higher in cities in models adjusted only for age and sex but this association largely disappeared in the fully adjusted models; these may represent over adjustment as both housing tenure and car ownership have a recognised urban/rural bias. The higher mental health treatment rates in cities may be a consequence of a greater availability of community mental health services or psychiatric facilities in different areas; though difference in the attitudes of rural residents should also be considered as they might have a preference for self-reliance and thus do not engage with health services for a mental health problem [48]. It is noteworthy that for individuals with chronic breathing problems there was no link between area of residence and the likelihood to receive appropriate treatment; which might support the presence of a complex interplay between rurality, access to services and stigma avoidance of mental healthcare services that does not apply to physical ill health.

### Limitations

This study has some significant strengths and limitations. It is a large population-based record-linkage study that utilised the 2011 Census, which provided prevalence estimates of chronic mental health status, and a centralised electronic database of prescribed medications, which included standardised categories of all medications prescribed by GPs and dispensed from community pharmacies across Northern Ireland. This approach obviated the selection biases inherent in many population surveys and provided an accurate and independent picture of treatment patterns. However, the use of the chronic mental health Census question might cause concern as it is obviously based on self-report, though the validity of the measure has been previously explored [27] and variations in mental ill health rates identified in this study, including the link between household and area deprivation, were in accordance with previous evidence on neighbourhood quality and mental health [49].

The study relies on receipt of medications prescribed by GPs and dispensed from community pharmacies, excluding medication prescribed in hospital settings, and this may have underestimated some further information biases as a small proportion of patients will have received a prescription but not have taken it to the pharmacist or not have used the dispensed medication. Further analysis into different psychotropic medication was limited by small numbers of individuals prescribed specific types medication for a more severe condition (such as schizophrenia). A notable omission is a lack of information on the use of non-pharmacological treatment such as the recommended Cognitive Behavioural Therapy [15]. Unfortunately, data on these treatment pathways were not readily available. However, the availability of these treatments is known to be limited in Northern Ireland and associated with significant delays [50], and in any event, it is recognised that even amongst these patients a high percentage will be also treated pharmacologically. We think it is likely that the inclusion of psychological therapies could perhaps have further accentuated rather than reduced the observed treatment patterns.

Finally, it is acknowledged that we do not have a true measure of need in terms of type or severity of ill health and therefore of the appropriateness of treatment. It is therefore possible that some of the differences between groups could represent over-treatment. Mitchell et al. [18] have indicated that GPs have a diagnostic sensitivity for depression of approximately 50% of “true cases” with false positives outnumbering missed cases, leading to more over-treatment than under-diagnosis [12].

### Implications

This study suggests that social and demographic factors are closely but moderately associated with the likelihood of treatment for poor mental health on a population level. However, overcoming the associated barriers in help-seeking behaviours will not be easy. Where access to primary care is likely to be an issue perhaps an increased awareness amongst GPs would increase the potential for case-finding amongst these infrequent attenders when they do eventually present at primary care. It is evident that much more needs to be done to reduce the stigma in society associated with mental health, but it is less clear what works. Notwithstanding that many public education and social marketing campaigns, such as the “Time to Change” campaign [51,52], have been implemented to reduce stigma and its impact on mental health help-seeking, it remains an obstacle to improving quality of life for people with mental ill health [53] and overcoming barriers among specific socio-demographic groups on a population level [54]. Perhaps these campaigns also need to be more targeted. Campaigns already recognise that there is a need to focus on men, (with examples such as Beyondblue [55] and Spur Projects [56]), but there is possibly a need for additional focus on ethnic minority sub-populations and on people living in rural communities. Finally, there is a need to better understand the conceptualisation, reporting and treatment preferences of the different ethnic minorities. Further qualitative studies should try to unbundle the obvious heterogeneity within this community and determine the role of language and knowledge of the processes of health services, as well as, the extent to which the current lower levels of medication represent a preference for alternative treatment approaches or true under-treatment.

## Conclusion

In this population-based study of individuals experiencing chronic mental ill health, the presence of social patterns in pharmacological treatment practices was identified as the likelihood of being in receipt of psychotropic medication varied by gender, ethnicity, marital and employment status. This study emphasises the need to focus on current prescribing practices and their link to healthcare inequalities across the population.

## Ethical approval

All procedures performed in studies involving human participants were in accordance with the ethical standards of the institutional and/or national research committee and with the 1964 Helsinki declaration and its later amendments or comparable ethical standards. For this type of study, formal consent is not required.

## Authors’ contributions

FT, DOR and MR were responsible for the design of the study. FT performed the analysis, supervised by MR and DOR. All authors contributed to the intellectual content of the paper as well as offering revisions on drafts.

## Abbreviations

BNF	British National Formulary
EPD	Enhanced Prescribing Database
GHQ	General Health Questionnaire
GPs	General Practitioners
NI	Northern Ireland
NILS	Northern Ireland Longitudinal Study
NISRA	Northern Ireland Statistics and Research Agency
NS-SEC	National Statistics Socio-economic Classification
SOA	Super Output Area

## Appendix

**Table 3: Proportions and characteristics of the people aged 25-74 who reported breathing difficulties at Census and the factors associated with the likelihood of receiving respiratory medical treatment in the following 12 months. Data represent numbers (percentages) and Odds Ratios (95% Confidence Interval) from logistic regression models.** $ fully adjusted model, adjusted for all variables listed in column one – age, sex, ethnicity, marital status, highest educational qualification, tenure, car access, locale & area-level deprivation measures

		Reported breathing problems at census	Received prescription in the 12 months following census	Adjusted: age & sex	Fully Adjusted^$^
		N(%)	N (%)	%	OR (95% CI)

Cohort		25,983 (9.1)	21,306 (82.0)

Age	25-34	3,386 (5.5)	1,210 (35.7)	1.00	1.00
	35-44	4,032 (6.1)	1,834 (45.5)	1.50 (1.37-1.65)	1.42 (1.29-1.57)
	45-54	5,421 (8.1)	2,712 (50.0)	1.80 (1.64-1.96)	1.62 (1.47-1.79)
	55-64	6,488 (12.3)	3,319 (51.2)	1.89 (1.74-2.06)	1.66 (1.51-1.83)
	65-74	6,656 (17.0)	3,407 (51.2)	1.89 (1.74-2.06)	1.64 (1.48-1.81)

Sex	Male	11,716 (8.5)	5,318 (45.4)	1.00	1.00
	Female	14,267 (9.6)	7,164 (50.2)	1.22 (1.16-1.28)	1.21 (1.15-1.28)

Ethnicity	White	25,823 (9.1)	12,419 (48.1)	1.00	1.00
	Non-white	160 (4.1)	63 (39.4)	0.76 (0.55-1.04)	0.78 (0.56-1.07)

Marital status	Married	13,737 (8.1)	6,781 (49.4)	1.00	1.00
	Never married	5,413 (7.6)	2,240 (41.4)	0.89 (0.83-0.95)	0.87 (0.81-0.94)
	Sep-Div-Wid	6,833 (15.0)	3,461 (50.7)	0.99 (0.93-1.05)	0.96 (0.90-1.02)

Education	Degree-level	5,436 (6.0)	2,436 (44.8)	1.00	1.00
	Intermediate	8,555 (7.4)	3,884 (45.4)	1.02 (0.96-1.10)	1.03 (0.95-1.11)
	No qualifications	11,992 (15.1)	6,162 (51.4)	1.14 (1.07-1.22)	1.15 (1.05-1.24)

Social class/Economic activity	Managerial/Prof	5,341 (6.2)	2,456 (46.0)	1.00	1.00
	Intermediate	3,821 (7.7)	1,810 (47.4)	1.01 (0.92-1.09)	0.99 (0.90-1.08)
	Own account	1,686 (7.5)	766 (45.4)	0.97 (0.87-1.09)	0.93 (0.83-1.05)
	Semi-routine	6,854 (10.3)	3,317 (48.4)	1.03 (0.96-1.11)	0.98 (0.90-1.06)
	Routine	5,477 (13.4)	2,817 (51.4)	1.13 (1.05-1.22)	1.05 (0.95-1.14)
	Never worked	2,137 (17.3)	1,037 (48.5)	1.02 (0.92-1.13)	0.94 (0.83-1.05)
	Unemployed	464 (7.4)	193 (41.6)	0.91 (0.75-1.10)	0.87 (0.72-1.07)
	Student	203 (8.0)	86 (42.4)	0.98 (0.74-1.31)	0.95 (0.71-1.27)

Housing tenure	Owner	16,537 (7.6)	7,923 (47.9)	1.00	1.00
	Renting	9,446 (13.6)	4,559 (48.3)	1.03 (0.98-1.08)	1.01 (0.95-1.08)
	Two or more cars	8,755 (6.1)	4,106 (46.9)	1.00	1.00

Household car access	One car	11,203 (10.7)	5,326 (48.3)	0.99 (0.94-1.05)	0.97 (0.91-1.04)
	No car	6,205 (16.0)	3,050 (49.2)	1.01 (0.95-1.08)	0.98 (0.90-1.07)

Locale of residence	Urban	10,481 (10.4)	5,086 (48.5)	1.00	1.00
	Intermediate	8,359 (9.3)	4,055 (48.5)	1.01 (0.96-1.07)	1.02 (0.96-1.09)
	Rural	4,545 (7.3)	2,134 (47.0)	0.96 (0.89-1.03)	0.98 (0.91-1.06)
	Missing	2,598 (7.5)	1,207 (46.5)	0.97 (0.89-1.06)	0.99 (0.90-1.08)

Area-level deprivation (SOA)	Least deprived	3,752 (6.7)	1,774 (47.3)	1.00	1.00
	Less deprived	4,559 (7.6)	2,114 (46.4)	0.96 (0.88-1.04)	0.95 (0.87-1.04)
	Average	4,760 (8.2)	2,260 (47.5)	1.00 (0.92-1.09)	0.99 (0.90-1.08)
	More deprived	5,628 (9.7)	2,705 (48.1)	1.01 (0.93-1.10)	0.99 (0.91-1.07)
	Most deprived	7,132 (13.5)	3,554 (49.8)	1.07 (0.99-1.16)	1.05 (0.96-1.15)
	Missing	152 (7.9)	75 (49.3)	1.03 (0.74-1.43)	1.04 (0.74-1.45)
